# Supplementary material for: Urate-lowering therapy may mitigate the risks of hospitalized stroke and mortality in patients with gout
Source: PLoS One. 2020 Jun 23;15(6):e0234909. doi: 10.1371/journal.pone.0234909 (PMC7310696; doi:10.1371/journal.pone.0234909)
Supplement: S1 Table — (DOCX) [file pone.0234909.s001.docx]

**S1 Table.** **Incidence and hazard ratio of hospitalized stroke between cohorts receiving or not receiving urate-lowering therapy.**

|  |  | Urate-lowering therapy | | | |  |  |  |  |
| --- | --- | --- | --- | --- | --- | --- | --- | --- | --- |
|  |  | No |  |  | Yes |  |  |  |  |
|  |  | Event | PY | IR | Event | PY | IR | cHR(95%CI) | aHR(95%CI) |
| Overall |  | 118 | 11326 | 1.0 | 74 | 12019 | 0.6 | 0.59(0.44,0.79)*** | 0.52(0.39,0.7)*** |
| XO inhibitors | | 118 | 11326 | 1.0 | 35 | 3607 | 1.0 | 0.93(0.64,1.36) | 0.7(0.47,1.03) |
| Uricosuric agents | | 118 | 11326 | 1.0 | 39 | 8412 | 0.5 | 0.44(0.31,0.64)*** | 0.42(0.29,0.61)*** |
| Sex |  |  |  |  |  |  |  |  |  |
| Female |  | 37 | 2574 | 1.4 | 29 | 2726 | 1.1 | 0.74(0.46,1.2) | 0.46(0.27,0.78)** |
| Male |  | 81 | 8752 | 0.9 | 45 | 9293 | 0.5 | 0.52(0.36,0.75)*** | 0.53(0.37,0.77)*** |
| Age, years | |  |  |  |  |  |  |  |  |
| 20-39 |  | 6 | 3935 | 0.2 | 5 | 4255 | 0.1 | 0.77(0.23,2.51) | 1.56(0.33,7.43) |
| 40-59 |  | 34 | 4968 | 0.7 | 18 | 5102 | 0.4 | 0.51(0.29,0.91)* | 0.46(0.25,0.83)** |
| 60-79 |  | 78 | 2423 | 3.2 | 51 | 2662 | 1.9 | 0.59(0.42,0.84)** | 0.55(0.38,0.8)** |
| Area |  |  |  |  |  |  |  |  |  |
| North |  | 47 | 5291 | 0.9 | 34 | 5730 | 0.6 | 0.67(0.43,1.04) | 0.66(0.42,1.05) |
| Central |  | 27 | 2338 | 1.2 | 17 | 2414 | 0.7 | 0.61(0.33,1.12) | 0.35(0.17,0.71)** |
| South |  | 42 | 3296 | 1.3 | 21 | 3473 | 0.6 | 0.47(0.28,0.8)** | 0.41(0.24,0.72)** |
| Other |  | 2 | 401 | 0.5 | 2 | 403 | 0.5 | 0.99(0.14,7.02) | 0.34(0.01,15.2) |
| Comorbidity | |  |  |  |  |  |  |  |  |
| Hypertension | No | 39 | 8246 | 0.5 | 22 | 8593 | 0.3 | 0.54(0.32,0.91)* | 0.59(0.34,1.02) |
|  | Yes | 79 | 3080 | 2.6 | 52 | 3426 | 1.5 | 0.59(0.42,0.84)** | 0.51(0.36,0.74)*** |
| DM | No | 91 | 10363 | 0.9 | 53 | 10901 | 0.5 | 0.55(0.39,0.77)*** | 0.51(0.36,0.72)*** |
|  | Yes | 27 | 963 | 2.8 | 21 | 1118 | 1.9 | 0.66(0.37,1.17) | 0.58(0.32,1.06) |
| CAD | No | 87 | 10169 | 0.9 | 57 | 10790 | 0.5 | 0.62(0.44,0.86)** | 0.58(0.41,0.81)** |
|  | Yes | 31 | 1157 | 2.7 | 17 | 1230 | 1.4 | 0.52(0.29,0.94)* | 0.32(0.16,0.63)** |
| Stroke | No | 85 | 10730 | 0.8 | 54 | 11309 | 0.5 | 0.6(0.43,0.85)** | 0.56(0.4,0.79)*** |
|  | Yes | 33 | 596 | 5.5 | 20 | 710 | 2.8 | 0.49(0.28,0.86)* | 0.43(0.24,0.79)** |
| Heart failure | No | 111 | 11155 | 1.0 | 68 | 11752 | 0.6 | 0.58(0.43,0.79)*** | 0.52(0.38,0.71)*** |
|  | Yes | 7 | 171 | 4.1 | 6 | 268 | 2.2 | 0.53(0.18,1.61) | 0.01(0,0.59)* |
| Hypercholesterolemia | No | 76 | 8557 | 0.9 | 42 | 8866 | 0.5 | 0.53(0.36,0.77)** | 0.51(0.35,0.75)*** |
|  | Yes | 42 | 2769 | 1.5 | 32 | 3154 | 1.0 | 0.65(0.41,1.03) | 0.52(0.32,0.84)** |
| Peripheral vascular diseases | No | 104 | 11078 | 0.9 | 71 | 11742 | 0.6 | 0.64(0.48,0.87)** | 0.58(0.43,0.79)*** |
|  | Yes | 14 | 248 | 5.6 | 3 | 278 | 1.1 | 0.18(0.05,0.64)** | 0.06(0.01,0.41)** |
| Atrial fibrillation | No | 113 | 11269 | 1.0 | 72 | 11938 | 0.6 | 0.6(0.45,0.81)*** | 0.53(0.4,0.72)*** |
|  | Yes | 5 | 57 | 8.8 | 2 | 81 | 2.5 | 0.2(0.04,1.06) |  |
| Rheumatologic diseases | No | 116 | 10994 | 1.1 | 72 | 11694 | 0.6 | 0.58(0.43,0.78)*** | 0.52(0.38,0.7)*** |
|  | Yes | 2 | 331 | 0.6 | 2 | 325 | 0.6 | 0.97(0.14,6.88) |  |
| Renal diseases | No | 112 | 11062 | 1.0 | 73 | 11675 | 0.6 | 0.62(0.46,0.83)** | 0.55(0.41,0.74)*** |
|  | Yes | 6 | 264 | 2.3 | 1 | 344 | 0.3 | 0.15(0.02,1.25) |  |
| Alcohol-related diseases | No | 110 | 11012 | 1.0 | 72 | 11664 | 0.6 | 0.62(0.46,0.83)** | 0.55(0.4,0.74)*** |
|  | Yes | 8 | 314 | 2.5 | 2 | 355 | 0.6 | 0.23(0.05,1.07) | 0.01(0,1.29) |
| Drug |  |  |  |  |  |  |  |  |  |
| ACE inhibitors/ARBs | No | 62 | 9536 | 0.7 | 25 | 9923 | 0.3 | 0.39(0.24,0.61)*** | 0.38(0.24,0.61)*** |
|  | Yes | 56 | 1790 | 3.1 | 49 | 2096 | 2.3 | 0.74(0.5,1.09) | 0.65(0.43,0.96)* |
| β-blockers | No | 45 | 8074 | 0.6 | 32 | 8483 | 0.4 | 0.67(0.43,1.06) | 0.65(0.41,1.04) |
|  | Yes | 73 | 3252 | 2.2 | 42 | 3536 | 1.2 | 0.53(0.36,0.77)** | 0.43(0.29,0.63)*** |
| Calcium-channel blockers | No | 41 | 8776 | 0.5 | 27 | 9121 | 0.3 | 0.63(0.39,1.03) | 0.65(0.39,1.06) |
|  | Yes | 77 | 2549 | 3.0 | 47 | 2898 | 1.6 | 0.53(0.37,0.76)*** | 0.47(0.32,0.68)*** |
| Diuretics | No | 60 | 9163 | 0.7 | 39 | 9430 | 0.4 | 0.63(0.42,0.94)* | 0.61(0.4,0.92)* |
|  | Yes | 58 | 2162 | 2.7 | 35 | 2590 | 1.4 | 0.5(0.33,0.77)** | 0.43(0.28,0.67)*** |
| Potassium sparing diuretics | No | 110 | 11108 | 1.0 | 70 | 11764 | 0.6 | 0.6(0.44,0.81)*** | 0.53(0.39,0.73)*** |
|  | Yes | 8 | 218 | 3.7 | 4 | 255 | 1.6 | 0.42(0.12,1.38) | 0.01(0,0.49)* |
| Other antihypertensive | No | 82 | 9989 | 0.8 | 45 | 10481 | 0.4 | 0.52(0.36,0.75)*** | 0.43(0.3,0.63)*** |
|  | Yes | 36 | 1337 | 2.7 | 29 | 1538 | 1.9 | 0.69(0.43,1.13) | 0.68(0.41,1.14) |
| Metformin | No | 101 | 10765 | 0.9 | 59 | 11346 | 0.5 | 0.55(0.4,0.76)*** | 0.51(0.37,0.7)*** |
|  | Yes | 17 | 561 | 3.0 | 15 | 673 | 2.2 | 0.74(0.37,1.49) | 0.75(0.36,1.58) |
| sulfonylurea | No | 98 | 10692 | 0.9 | 56 | 11278 | 0.5 | 0.54(0.39,0.75)*** | 0.48(0.35,0.67)*** |
|  | Yes | 20 | 634 | 3.2 | 18 | 741 | 2.4 | 0.76(0.4,1.44) | 0.79(0.4,1.56) |
| Insulin | No | 109 | 11125 | 1.0 | 66 | 11759 | 0.6 | 0.57(0.42,0.78)*** | 0.51(0.37,0.69)*** |
|  | Yes | 9 | 201 | 4.5 | 8 | 260 | 3.1 | 0.66(0.25,1.71) | 1.43(0.31,6.49) |
| Statin | No | 98 | 10636 | 0.9 | 61 | 11137 | 0.6 | 0.59(0.43,0.82)** | 0.57(0.41,0.78)*** |
|  | Yes | 20 | 690 | 2.9 | 13 | 882 | 1.5 | 0.52(0.26,1.06) | 0.33(0.14,0.75)** |
| Aspirin | No | 76 | 9736 | 0.8 | 46 | 10305 | 0.5 | 0.57(0.4,0.82)** | 0.55(0.38,0.8)** |
|  | Yes | 42 | 1590 | 2.6 | 28 | 1714 | 1.6 | 0.6(0.37,0.97)* | 0.43(0.26,0.73)** |

IR, incidence rate, per 100 person-years; PY, person-years; CI, confidence interval; cHR, crude hazard ratio; aHR, adjusted hazard ratio, controlling for sex, age, area, every comorbidity, and drug in Table 1; XO inhibitors, xanthine oxidase inhibitors, consisting of allopurinol and febuxostat; Uricosuric agents, consisting of benzbromarone, probenecid, and sulfinpyrazone; * *p*<0.05, ** *p*<0.01, *** *p*<0.001.
