# Supplementary material for: Urate-lowering therapy may mitigate the risks of hospitalized stroke and mortality in patients with gout
Source: PLoS One. 2020 Jun 23;15(6):e0234909. doi: 10.1371/journal.pone.0234909 (PMC7310696; doi:10.1371/journal.pone.0234909)
Supplement: S2 Table — (DOCX) [file pone.0234909.s002.docx]

**S2Table.** **Incidence and hazard ratio of all-cause mortality between cohorts receiving or not receiving urate-lowering therapy.**

|  |  | Urate-lowering therapy | | | |  |  |  |  |
| --- | --- | --- | --- | --- | --- | --- | --- | --- | --- |
|  |  | No |  |  | Yes |  |  |  |  |
|  |  | Event | PY | IR | Event | PY | IR | cHR(95%CI) | aHR(95%CI) |
| Overall |  | 57 | 11745 | 0.5 | 37 | 12233 | 0.3 | 0.62(0.41,0.94)* | 0.6(0.39,0.92)* |
| XO inhibitors | | 57 | 11745 | 0.5 | 8 | 3712 | 0.2 | 0.44(0.21,0.92)* | 0.35(0.17,0.75)** |
| Uricosuric agents | | 57 | 11745 | 0.5 | 29 | 8520 | 0.3 | 0.7(0.45,1.1) | 0.75(0.47,1.18) |
| **Sex** |  |  |  |  |  |  |  |  |  |
| Female |  | 11 | 2715 | 0.4 | 10 | 2829 | 0.4 | 0.88(0.37,2.07) | 0.77(0.29,2.04) |
| Male |  | 46 | 9031 | 0.5 | 27 | 9403 | 0.3 | 0.56(0.35,0.91)* | 0.59(0.37,0.96)* |
| Age, years | |  |  |  |  |  |  |  |  |
| 20-39 |  | 7 | 3949 | 0.2 | 4 | 4270 | 0.1 | 0.52(0.15,1.79) | 0.48(0.12,1.88) |
| 40-59 |  | 19 | 5107 | 0.4 | 12 | 5157 | 0.2 | 0.62(0.3,1.29) | 0.53(0.24,1.18) |
| 60-79 |  | 31 | 2689 | 1.2 | 21 | 2806 | 0.8 | 0.67(0.38,1.16) | 0.58(0.32,1.03) |
| Area |  |  |  |  |  |  |  |  |  |
| North |  | 24 | 5461 | 0.4 | 24 | 5842 | 0.4 | 0.93(0.53,1.63) | 0.92(0.51,1.65) |
| Central |  | 13 | 2422 | 0.5 | 6 | 2462 | 0.2 | 0.46(0.17,1.2) | 0.24(0.07,0.85)* |
| South |  | 20 | 3456 | 0.6 | 5 | 3525 | 0.1 | 0.26(0.1,0.69)** | 0.21(0.07,0.63)** |
| Other |  | 0 | 407 | 0.0 | 2 | 403 | 0.5 |  |  |
| Comorbidity | |  |  |  |  |  |  |  |  |
| Hypertension | No | 30 | 8380 | 0.4 | 15 | 8643 | 0.2 | 0.48(0.26,0.9)* | 0.43(0.23,0.83)* |
|  | Yes | 27 | 3365 | 0.8 | 22 | 3589 | 0.6 | 0.76(0.43,1.33) | 0.63(0.34,1.14) |
| DM | No | 52 | 10642 | 0.5 | 30 | 11065 | 0.3 | 0.55(0.35,0.86)** | 0.54(0.34,0.85)** |
|  | Yes | 5 | 1103 | 0.5 | 7 | 1168 | 0.6 | 1.26(0.4,3.99) | 1.63(0.37,7.08) |
| CAD | No | 43 | 10484 | 0.4 | 25 | 10958 | 0.2 | 0.55(0.34,0.91)* | 0.6(0.36,1)* |
|  | Yes | 14 | 1261 | 1.1 | 12 | 1274 | 0.9 | 0.83(0.38,1.8) | 0.58(0.24,1.39) |
| Stroke | No | 42 | 11040 | 0.4 | 32 | 11450 | 0.3 | 0.73(0.46,1.15) | 0.7(0.44,1.12) |
|  | Yes | 15 | 705 | 2.1 | 5 | 782 | 0.6 | 0.31(0.11,0.86)* | 0.18(0.06,0.59)** |
| Heart failure | No | 50 | 11548 | 0.4 | 35 | 11951 | 0.3 | 0.67(0.44,1.04) | 0.68(0.44,1.05) |
|  | Yes | 7 | 198 | 3.5 | 2 | 281 | 0.7 | 0.18(0.04,0.86)* |  |
| Hypercholesterolemia | No | 44 | 8813 | 0.5 | 24 | 8995 | 0.3 | 0.54(0.33,0.88)* | 0.51(0.31,0.85)* |
|  | Yes | 13 | 2933 | 0.4 | 13 | 3238 | 0.4 | 0.9(0.41,1.93) | 0.74(0.31,1.78) |
| Peripheral vascular diseases | No | 52 | 11444 | 0.5 | 34 | 11952 | 0.3 | 0.62(0.4,0.96)* | 0.63(0.4,0.97)* |
|  | Yes | 5 | 301 | 1.7 | 3 | 281 | 1.1 | 0.63(0.15,2.67) |  |
| Atrial fibrillation | No | 54 | 11679 | 0.5 | 37 | 12147 | 0.3 | 0.66(0.43,1)* | 0.65(0.42,0.99)* |
|  | Yes | 3 | 67 | 4.5 | 0 | 86 | 0.0 |  |  |
| Rheumatologic diseases | No | 54 | 11403 | 0.5 | 33 | 11902 | 0.3 | 0.58(0.38,0.9)* | 0.56(0.36,0.87)** |
|  | Yes | 3 | 342 | 0.9 | 4 | 330 | 1.2 | 1.4(0.31,6.27) |  |
| Renal diseases | No | 49 | 11472 | 0.4 | 36 | 11888 | 0.3 | 0.71(0.46,1.09) | 0.7(0.45,1.08) |
|  | Yes | 8 | 274 | 2.9 | 1 | 345 | 0.3 | 0.08(0.01,0.68)* |  |
| Alcohol-related diseases | No | 52 | 11415 | 0.5 | 29 | 11873 | 0.2 | 0.53(0.34,0.84)** | 0.52(0.33,0.83)** |
|  | Yes | 5 | 330 | 1.5 | 8 | 359 | 2.2 | 1.49(0.48,4.57) | 1.81(0.21,15.5) |
| Drug |  |  |  |  |  |  |  |  |  |
| ACE inhibitors/ARBs | No | 36 | 9753 | 0.4 | 17 | 9981 | 0.2 | 0.46(0.26,0.82)** | 0.45(0.25,0.82)** |
|  | Yes | 21 | 1992 | 1.1 | 20 | 2252 | 0.9 | 0.85(0.46,1.56) | 0.75(0.39,1.43) |
| β-blockers | No | 27 | 8219 | 0.3 | 19 | 8564 | 0.2 | 0.67(0.37,1.21) | 0.6(0.32,1.12) |
|  | Yes | 30 | 3526 | 0.9 | 18 | 3668 | 0.5 | 0.58(0.32,1.04) | 0.51(0.28,0.94)* |
| Calcium-channel blockers | No | 30 | 8919 | 0.3 | 14 | 9181 | 0.2 | 0.45(0.24,0.85)* | 0.51(0.27,0.98)* |
|  | Yes | 27 | 2827 | 1.0 | 23 | 3052 | 0.8 | 0.79(0.45,1.38) | 0.69(0.38,1.24) |
| Diuretics | No | 34 | 9401 | 0.4 | 22 | 9547 | 0.2 | 0.64(0.37,1.09) | 0.58(0.33,1.02) |
|  | Yes | 23 | 2345 | 1.0 | 15 | 2685 | 0.6 | 0.57(0.29,1.08) | 0.49(0.24,0.99)* |
| Potassium sparing diuretics | No | 50 | 11501 | 0.4 | 35 | 11968 | 0.3 | 0.67(0.44,1.03) | 0.65(0.42,1.01) |
|  | Yes | 7 | 244 | 2.9 | 2 | 265 | 0.8 | 0.27(0.06,1.32) |  |
| Other antihypertensive | No | 41 | 10268 | 0.4 | 22 | 10603 | 0.2 | 0.51(0.31,0.86)* | 0.49(0.29,0.84)** |
|  | Yes | 16 | 1478 | 1.1 | 15 | 1629 | 0.9 | 0.88(0.44,1.79) | 0.76(0.36,1.64) |
| Metformin | No | 51 | 11107 | 0.5 | 30 | 11523 | 0.3 | 0.56(0.36,0.88)* | 0.55(0.35,0.87)* |
|  | Yes | 6 | 639 | 0.9 | 7 | 710 | 1.0 | 1.07(0.36,3.19) | 1.37(0.29,6.46) |
| sulfonylurea | No | 51 | 11018 | 0.5 | 30 | 11447 | 0.3 | 0.56(0.36,0.88)* | 0.55(0.35,0.87)* |
|  | Yes | 6 | 727 | 0.8 | 7 | 785 | 0.9 | 1.07(0.36,3.2) | 1.48(0.37,5.96) |
| Insulin | No | 54 | 11506 | 0.5 | 34 | 11950 | 0.3 | 0.6(0.39,0.93)* | 0.56(0.36,0.87)* |
|  | Yes | 3 | 240 | 1.3 | 3 | 282 | 1.1 | 0.84(0.17,4.17) | 0.64(0.01,50.2) |
| Statin | No | 53 | 10970 | 0.5 | 32 | 11316 | 0.3 | 0.58(0.38,0.9)* | 0.57(0.36,0.89)* |
|  | Yes | 4 | 775 | 0.5 | 5 | 917 | 0.6 | 1.05(0.28,3.94) |  |
| Aspirin | No | 40 | 10001 | 0.4 | 27 | 10451 | 0.3 | 0.64(0.39,1.04) | 0.66(0.4,1.1) |
|  | Yes | 17 | 1744 | 1.0 | 10 | 1782 | 0.6 | 0.57(0.26,1.24) | 0.41(0.17,1) |

IR, incidence rate, per 100 person-years; PY, person-years; CI, confidence interval; cHR, crude hazard ratio; aHR, adjusted hazard ratio, controlling for sex, age, area, every comorbidity, and drug in Table 1; XO inhibitors, xanthine oxidase inhibitors, consisting of allopurinol and febuxostat; Uricosuric agents, consisting of benzbromarone, probenecid, and sulfinpyrazone; * *p*<0.05, ** *p*<0.01, *** *p*<0.001.
