# Supplementary material for: Urate-lowering therapy may mitigate the risks of hospitalized stroke and mortality in patients with gout
Source: PLoS One. 2020 Jun 23;15(6):e0234909. doi: 10.1371/journal.pone.0234909 (PMC7310696; doi:10.1371/journal.pone.0234909)
Supplement: S3 Table — (DOCX) [file pone.0234909.s003.docx]

**S3 Table.** **Cox model measured hazard ratios of hospitalized stroke associated with variables restricted on uricosuric agents.**

| Variable | Event | PY | IR | cHR(95%CI) | aHR(95%CI) |
| --- | --- | --- | --- | --- | --- |
| Matched cohort | |  |  |  |  |
| Non-users (n=2609) | 118 | 11326 | 1.0 | 1(ref) | 1(ref) |
| Users (n=1840) | 39 | 8412 | 0.5 | 0.44(0.31,0.64)*** | 0.42(0.29,0.61)*** |
| Cumulative duration of therapy (months) | | | | |  |
| Non-users | 118 | 11326 | 1.0 | 1(ref) | 1(ref) |
| ≦1 | 9 | 1976 | 0.5 | 0.44(0.22,0.86)* | 0.5(0.25,0.99)* |
| 1-5 | 14 | 2744 | 0.5 | 0.49(0.28,0.85)* | 0.56(0.32,0.98)* |
| >5 | 16 | 3692 | 0.4 | 0.42(0.25,0.7)*** | 0.31(0.18,0.53)*** |
| *p* for trend |  |  |  | <.001 | <.001 |
| Monthly Average daily doses of urate-lowering therapy | | | | | |
| Non-users | 118 | 11326 | 1.0 | 1(ref) | 1(ref) |
| ≦0.5 | 17 | 2916 | 0.6 | 0.56(0.34,0.93)* | 0.45(0.27,0.77)** |
| 0.5-0.8 | 8 | 1874 | 0.4 | 0.41(0.2,0.84)* | 0.38(0.18,0.78)** |
| >0.8 | 14 | 3622 | 0.4 | 0.37(0.21,0.65)*** | 0.39(0.22,0.68)*** |
| *p* for trend |  |  |  | <.001 | <.001 |

IR, incidence rate, per 100 person-years; PY, person-years; CI, confidence interval; cHR, crude hazard ratio; aHR, adjusted hazard ratio, controlling for sex, age, area, every comorbidity, and drug in Table 1; * *p*<0.05, ** *p*<0.01, ****p* <0.001.
