# Supplementary material for: Urate-lowering therapy may mitigate the risks of hospitalized stroke and mortality in patients with gout
Source: PLoS One. 2020 Jun 23;15(6):e0234909. doi: 10.1371/journal.pone.0234909 (PMC7310696; doi:10.1371/journal.pone.0234909)
Supplement: Attachment — Submitted filename: Response Letter PLOS ONE ULT AND STROKE Hsu.doc [file pone.0234909.s005.doc]

Hans-Peter Brunner-La Rocca, M.D.

Academic Editor

PLOS ONE

March 23, 2020

Dear Dr. Hans-Peter Brunner-La Rocca:

Re: Document reference No. PONE-D-19-31773

Please find attached a revised version of our document “Urate-lowering therapy may mitigate the risks of hospitalized stroke and mortality in patients with gout”. We would like to resubmit for publication as an original article in *PLOS ONE*.

Your comments and those of the reviewers were highly insightful and enabled us to improve the quality of our document. In the following pages are our responses to each comment from the reviewer as well as your own comments.

Revisions in the text are shown with tracked changes. We hope that our revisions to the document combined with our accompanying responses will be sufficient to render our document suitable for publication in *PLOS ONE*.

We look forward to hearing from you soon.

Yours sincerely,

Chii-Min Hwu

Faculty of Medicine, National Yang-Ming University School of Medicine, and Section of Endocrinology and Metabolism, Department of Medicine, Taipei Veterans General Hospital

Tel.: +886 2 28757516

Fax: +886 2 28751429

E-Mail: chhwu@vghtpe.gov.tw

Address: No. 201, Sec. 2 Shi-Pai Rd., Chung-Cheng Build. 11F Room522, Taipei 112, Taiwan.

**Responses to the comments of Editor**

1. The authors should address the differences between stroke reduction and effects on CAD and HF in more detail. In other words, why do they think that different effects were seen? Is this pure coincidence or is there some specific rational possibly responsible for this? To me, it may be an indication for significant co-founding factors that could not be taken into account, indicating the limitation of the analysis present. The authors addressed some limitation in this regard, but I would like to see some discussion about the differences mentioned above.

*Response: Thank you for your suggestions, we added the mention at lines243-245 as: “Previous studies indicated that ULT may reduce blood pressure [8,9], blood pressure has a greater impact on stroke than on heart diseases [26]; which may explain our results that ULT associated with lower risk of stroke and with no significant risk of CAD and heart failure. “*

2. The authors should be much more cautious about their conclusion. This is a retrospective study, matching was limited as many factors were not available, there was other chance of bias as indicated by the authors. Together, this means that the study is not more than hypothesis generating and the authors should be clear on this.

*Response:* *Thank you for your suggestions! We modified our conclusions at lines 45-46, 203-204, 293-294 as” Our study revealed that ULT was associated with lower risks of hospitalized stroke and all-cause mortality compared with the absence of ULT in patients with gout.”*

3. I agree with the reviewer that the discussion could be shortened significantly despite the request for additional thoughts to be discussed. The previous findings of retrospective analyses should be summarized and less extensive information in this regard is required.

*Response: We tried to shorten the discussion, summarize previous analytic findings with less extensive information, and added the requested thoughts in the discussions.*

4. Please present the tables in a way that they can be easily read.

*Response:* *We corrected the tables 1 and 2 to let them be easily read.*

**Responses to the comments of Reviewer 1**

1. The authors go to great lengths to propensity-match the urate lowering therapy users vs non-users and are to be commended. However, these efforts still obscure a deeper question: why were some individuals not being prescribed urate lowering therapy for established gout? Did they have milder gout, such that they did not meet criteria for treatment (for example, under American College of Rheumatology treatment guidelines, which specify that most patients should receive urate lowering therapy if they have ≥2 gout flares/year)? If they did have milder gout, would that affect the outcomes? Or did they have lower serum urate levels, such that their physicians did not feel compelled to add a urate lowering therapy? If so, would that bias the outcomes? Is it possible their

physicians were less conscientious than the physicians who did prescribed urate lowering therapy, and could the cardiovascular outcomes be a result of physician attention or inattention rather than the effect of the urate lowering drug itself? Or did the patients not receive urate lowering therapy because they themselves were less compliant with medical care, putting them at a more general risk for adverse cardiovascular outcomes? These questions need to be addressed.

*Response: Thank you for your reminding! It may be because most patients went to the outpatient clinics as an urgent gout attack; sometimes, the patients couldn’t be checked uric acid levels without fasting. In Taiwan, without recent (6 months) data of uric acid levels, the doctor can’t prescribe ULT to their gout patients. It is also possible that the patients did not come back for the uric acid test. The less compliance of patients to testing uric acid or taking urate lowering agents may further put them at a higher risk of adverse cardiovascular events. The milder gout of patients or the attention/inattention of physicians might also influence the prescribing of urate-lowering therapy. We added these limitations in the section of discussions at lines 284-286” Patients may have lower uric acid levels, so physicians don’t prescribe ULT for them. Without confirmation of uric acid or crystal identification, patients with some forms of arthritis may be misdiagnosed as gout. These potential biases also require attention.” and at lines 276-280 “However, patients with milder gout or less recurrent gouty attack may make physicians not feel compelled to prescribe ULT for them. Some patients may not come back for uric acid tests, or have poor adherence to urate-lowering medications; these confounding factors may influence our assessed outcomes. “*

2. The Discussion is way too long—it should be cut by 30-50%. However, it also is lacking certain important elements. For instance, I note that the entire discussion centers around the impact of urate, and urate-lowering, on cardiovascular disease. While this concern is appropriate, it totally misses the point that this is a study not one of asymptomatic hyperuricemics, but of gout patients who have additional issues besides hyperuricemia (e.g., intermittent inflammatory attacks). This warrants discussion. At least from a point of view of rigorous logic, the question of whether urate-lowering therapy will reduce cardiovascular events in patients with hyperuricemia without gout must remain speculative.

*Response:* *Thank you for your suggestions. We have shortened about 40% of the discussion with tracked changes; and added the statement about gout with inflammatory attack at lines 215-217” 3. Gout, with intermittent inflammatory attacks, also exerts increased risks of morbidity and mortality from CV diseases [20].” We also added the limitation at lines 290-291 “Fifth, because we selected patients with gout, our results may not be applicable to hyperuricemic patients without gouty attack.” to make our manuscript clearer.*

3. The lack of available serum urate levels is a problem that deserves more attention in the text. First of all, did the patients who received urate lowering have higher urate levels at the start than those in the not urate-lowering group? Second, were the urate-lowering drugs actually successful in lowering urate—which might have provided insight into compliance with the medications as well as their mechanism of cardiovascular effect? It is surprising that serum urate levels were not available in patients getting urate lowering therapy

*Response: It is a pity that the administrative dataset is lack of serum urate levels. We have added this limitation at lines 282-287 “Third, we could not obtain the blood pressure levels of patients or their biochemical blood test results, such as UA, blood glucose, HBA1C, cholesterol, LDL, and creatinine. Patients may have lower uric acid levels, so physicians don’t prescribe ULT for them. Without confirmation of uric acid or crystal identification, patients with some forms of arthritis may be misdiagnosed as gout. These potential biases also require attention.”*

4. While not at all the fault of the investigators, the data has inconsistencies that deserve more comment. For example, how is it possible to have more all-cause mortality (presumably hospitalized cardiovascular death), without also having cardiovascular death? Are there other, perhaps unanticipated causes of death that are reduced with urate lowering?

*Response: Our study revealed that* *ULT in patients with gout was associated with lower risks of hospitalized stroke and all-cause mortality (Table 2, page 16). However, the decreased magnitude of hospitalized stroke by ULT may not be big enough to significantly reduce the risk of CV death.*

5. It is interesting that the impact of urate lowering turned out not to be a generalized phenomenon, but was specific to the drug in question. If two drugs that lower urate by different mechanisms have different and non-overlapping protective effects (i.e., uricosurics reducing stroke, and xanthine oxidase inhibitors reducing all-cause mortality), how can one conclude that both are due to the same outcome of urate lowering and not other, unrecognized, divergent effects? This deserves additional consideration.

Response: We agree with your opinions. We changed the statement at lines 240-242 to describe the different mechanisms of uricosuric agents in reducing stroke” which might indicate that this protective effect occurs through the increasing excretion of SUA and not through the decrease in XO activity.”. We modified the statement at lines 256-258 as” which might indicate that the protection of death occurs through the reduction of xanthine oxidase activity and pro-oxidants, not solely by increasing excretion of uric acid.” By adding this statement, we provide additional consideration for the different mechanisms of xanthine oxidase inhibitors in reducing all-cause mortality.

6. The authors twice mention that no prior study has addressed the ability of urate lowering to reduce the development or progression of stroke. The recently-published FREED trial looked at urate-lowering with febuxostat versus a control group, and found an overall decrease in a composite outcome (cerebrovascular, cardiovascular and renal events and all deaths), but found no decrease with febuxostat (despite urate lowering) in cardiovascular events. This study should be noted.

*Response: Thank you for your suggestions! We deleted the statement of “no prior study has addressed the ability of urate lowering to reduce the development or progression of stroke.” and changed it (at line 238-239) into” Our study demonstrated that ULT might reduce the risk of hospitalized stroke”. We added the statement about Freed trial at lines 224-228” The Febuxostat for Cerebral and CaRdiorenovascular Events PrEvention StuDdy (FREED) trial compared febuxostat with a control group [22], and demonstrated a significant reduction of the composite outcome (cerebral, cardiovascular and renal events and all deaths); but found no decrease in cardiovascular events.” to make our discussion more complete.*

7. Can the authors clarify—it looks like the patients were censored after a single defined cardiovascular event. Is this correct? So then, subsequent events in the same patient (of a similar or different type) were not counted? This deserves to be explicitly mentioned.

*Response: Thank you! We clarified the definition of outcomes by adding statements at line 135-137 as “and the patients were censored after a single defined cardiovascular even*t. Separate Cox models were conducted to evaluate effects of ULT for different outcomes.”

8. The manuscript title is a bit inaccurate, since it addresses the reduction in stroke but not all cause mortality that the authors observed.

*Response: Thank for your reminding! We have changed the title as” Urate-lowering therapy may mitigate the risks of hospitalized stroke and mortality in patients with gout”.*
