# Supplementary material for: Urate-lowering therapy may mitigate the risks of hospitalized stroke and mortality in patients with gout
Source: PLoS One. 2020 Jun 23;15(6):e0234909. doi: 10.1371/journal.pone.0234909 (PMC7310696; doi:10.1371/journal.pone.0234909)
Supplement: Attachment — Submitted filename: O-2019-009200 R3 Response Letter.doc [file pone.0234909.s006.doc]

Hans-Peter Brunner-La Rocca, M.D.

Academic Editor

*PLOS ONE*

May 4, 2020

Dear Dr. Hans-Peter Brunner-La Rocca:

Re: Document reference No. PONE-D-19-31773R2

Please find attached a revised version of our document “Urate-lowering therapy may mitigate the risks of hospitalized stroke and mortality in patients with gout”. We would like to resubmit for publication as an original article in *PLOS ONE*.

Your comments and those of the reviewers were highly insightful and enabled us to improve the quality of our document. In the following pages are our responses to each comment from the reviewer as well as your own comments.

Revisions in the text are shown with tracked changes. We hope that our revisions to the document combined with our accompanying responses will be sufficient to render our document suitable for publication in *PLOS ONE*.

We look forward to hearing from you soon.

Yours sincerely,

Chii-Min Hwu

Faculty of Medicine, National Yang-Ming University School of Medicine, and Section of Endocrinology and Metabolism, Department of Medicine, Taipei Veterans General Hospital

Tel.: +886 2 28757516

Fax: +886 2 28751429

E-Mail: chhwu@vghtpe.gov.tw

Address: No. 201, Sec. 2 Shi-Pai Rd., Chung-Cheng Build. 11F Room522, Taipei 112, Taiwan.

**Responses to the comments of Editor**

1. As you can see in the comment by the reviewer, there are remaining issues. It is important that the interpretation of data is not speculative, unless clearly stated as such or stated as hypothesis that needs further tested.

*Response: We have gone through the whole paper and corrected the interpretation at page 3, 18, 19, 20 and 21 of the tracked manuscript to make it not speculative.*

2. In addition, I agree with the reviewer that the discussion should be shortened.

*Response:* *We tried to shorten the discussion by using summary sentence with multiple references at page 19, 20 and 21 to make the mention more concise.*

**Responses to the comments of Reviewer 1**

1. I thank the authors for their responses and resubmission. The manuscript is improved, but

unfortunately, I do not yet feel that the authors have fully responded to this reviewer’s comments. In my opinion, two specific issues remain to be addressed. There continues to persist language that implies causality between ULT and benefit in the interpretation of the data. It is fine to discuss possible causality in sections where mechanisms are being speculated about, but not in sections where the meaning of the data should be interpreted rigorously. Just as one example, at the beginning of

the Discussion the authors now appropriately state that “ULT in patients with gout was ASSOCIATED with lower risks of hospitalized stroke and all-cause mortality.” However, in the very next sentence they continue to state that “A subgroup analysis revealed that uricosuric agents HAVE A SIGNIFICANT EFFECT on the decrease of hospitalized stroke, and XO inhibitors HAVE A BENEFICIAL EFFECT on survival.” This causal statement is simply not the case from the data; instead the term “association” should be applied here as it is in the first sentence. The authors need to go through the manuscript and strip any inference of causality out of the interpretation of their data; they may leave such inferences in more speculative sections where they are discussing the possible meaning of their data.

*Response: Thank you for your encouragement. We have gone through the whole paper and corrected the interpretation at page 3, 18, 19, 20 and 21 of the tracked manuscript to make it not speculative.*

2. The discussion is still too long! It is 9 paragraphs and no reader will have patience for that. Much of the excessive content comes from the heaping up of examples that could be reduced to a summary sentence with multiple references, which would shorten the manuscript significantly. Honestly, there are very few good reasons for a discussion that focuses on the interpretation of a study to go more than 4 or 5 paragraphs.

*Response: We learned much from your recommendations. We have shortened the discussion by using summary sentence with multiple references at page 19, 20 and 21 to make the mention more concise.*
